# Deep-sea benthic crustacean and annelid data from the Bering Sea

**DOI:** 10.1016/j.dib.2023.109186

**Published:** 2023-04-28

**Authors:** Henry Knauber, Katharina Kohlenbach, Philipp Böhm, Carsten Lüter, Alexander Ziegler, Angelika Brandt, Hanieh Saeedi

**Affiliations:** a Department of Marine Zoology, Senckenberg Research Institute and Natural History Museum; Senckenberganlage 25, Frankfurt am Main 60325, Germany; b Diversity and Evolution, Goethe University Frankfurt, Institute for Ecology; Max-von-Laue-Straße 13, Frankfurt am Main 60438, Germany; c Natural History Museum, Leibniz Institute for Evolution and Biodiversity Science; Invalidenstraße 43, Berlin 10115, Germany; d Institute of Evolutionary Biology and Ecology, University of Bonn; An der Immenburg 1, Bonn 53121, Germany

**Keywords:** Abyssal, Benthos, Biogeography, Crustacea, Annelida, Sipuncula, Hirudinea, Polychaeta

## Abstract

Samples of Crustacea and Annelida (Polychaeta, Sipuncula, and Hirudinea) were collected in the Bering Sea and the northwestern Pacific Ocean during scientific cruise SO-249 BERING in 2016. Biological samples were collected from 32 locations by the team on-board RV Sonne using a chain bag dredge at depths ranging between 330–5,070 m, and preserved in 96% ethanol. Specimens were morphologically identified to the lowest taxonomic level possible using a Leica M60 stereomicroscope. The generated data here comprise taxonomic information as well as annotated bathymetric and biogeographic information from a total of 78 samples (26 Crustacea, 47 Polychaeta, 4 Sipuncula, and 1 Hirudinea). The dataset was prepared following Darwin Core Biodiversity standards for FAIR data sharing based on Ocean Biodiversity Information System (OBIS) and Global Biodiversity Facility (GBIF) guidelines. The standardised digitised data were then mobilised to both OBIS and GBIF under CC BY 4.0 licence to publicly share and adopt the data. As records of these important marine taxa from bathyal and abyssal depths are sparse, especially from the deep Bering Sea, the herein generated and digitised data aid in filling existing knowledge gaps on their diversity and distribution in that region. As part of the “Biogeography of the NW Pacific deep-sea fauna and their possible future invasions into the Arctic Ocean” (BENEFICIAL) project, this dataset thus not only increases our knowledge in re-assessing and uncovering the deep-sea diversity of these taxa, but also serves policy and management sectors by providing first-hand data for global report assessments.

**Specifications Table**

**Table:** 

Subject	Biodiversity
Specific subject area	Benthic deep-sea species diversity and biogeography
Type of data	Table
	Figure
	Occurrence, event, and ENV-DATA derived data based on Darwin Core standard [1]
How the data were acquired	Specimens were collected during the deep-sea expedition SO-249 BERING by dredging the greater Bering Sea region. Morphological studies of deep-sea taxa from biological samples were performed using a Leica M60 stereomicroscope. Species were identified using taxonomic keys and taxonomic literature.
Data format	Raw
Description of data collection	Unpublished (raw) data were collected by morphologically identifying ethanol-fixed specimens (Crustacea, Polychaeta, Sipuncula, Hirudinea) to the lowest taxonomic level possible from biological samples collected with a chain bag dredge during the deep-sea expedition SO-249 BERING in 2016.
Data source location	Institution: Museum für Naturkunde Berlin (ZMB)
	City: Berlin
	Country: Germany
	Samples were collected from the deep Bering Sea and the northwestern Pacific Ocean around the Aleutian Islands and Aleutian Trench. Details on data sources are listed in the cruise report [2].
Data accessibility	Repository name(s): Ocean Biodiversity Information System (OBIS); Global Biodiversity Information Facility (GBIF)
	Data identification numbers: (1) e0ce9f82-687e-4cbf-95d0-d27d1054bfe9
	(2) a0eebab0-5c66-449f-a9b2-00dc5a83f356
	Direct URL to data: (1) https://portal.obis.org/dataset/e0ce9f82-687e-4cbf-95d0-d27d1054bfe9[3]
	(2) https://www.gbif.org/dataset/a0eebab0-5c66-449f-a9b2-00dc5a83f356[4]
	The data are shared under CC BY 4.0 license as open-access data.
	Instructions for accessing the data: (1) Downloading the data on OBIS under “Source DwC-A”.
	(2) Downloading the data on GBIF under “Download” as “GBIF annotated archive”.
Related research article	Knauber, H., Kohlenbach, K., Brandt, A., & Saeedi, H. (2023). Crustaceans of the Northwest Pacific Ocean: Species richness and distribution patterns. J. Sea Res. 191, 102332. DOI: https://doi.org/10.1016/j.seares.2022.102332

## Value of the Data

• The absence of these raw records of deep-sea crustacean and annelid occurrences from the Bering Sea and the northwestern Pacific Ocean, particularly at bathyal and abyssal depths, constitutes a knowledge gap. Thus, the digitization of the present information adds valuable occurrence and diversity data for these taxa, allowing researchers to reassess their species richness patterns in that region.
• The data generated here further provide information needed to predict potential distribution range shifts of these taxa as a result of future climate change and growing anthropogenic stresses.
• In addition to providing information for data scientists and ecologists looking to analyse large-scale biodiversity and distribution patterns, the occurrence records included in this dataset aid taxonomists in understanding the patterns of distribution of the taxa herein described.
• In order to protect the deep sea as a vulnerable ecosystem from anthropogenic activities like deep-sea mining and climate change-induced alterations, this data can be used in policy-making processes, including providing data for deep-sea biodiversity assessment reports and stewardships.

## 1 Objective

As contribution to and part of the “Biogeography of the NW Pacific deep-sea fauna and their possible future invasions into the Arctic Ocean”-Project (BENEFICIAL; [6,7]), these data aid in (i) uncovering the distribution and diversity patterns of these taxa in the northwestern Pacific and (ii) potential shifts of key species into the Arctic Ocean due to the anthropogenic climate change and a potential invasion of alien species from the lower to the higher latitudes.

The crustacean records of the herein described dataset add valuable biodiversity data of otherwise poorly studied marine regions of the northwest Pacific Ocean, that has been studied in regard to crustacean biodiversity patterns in Knauber et al. [5], thus complementing the analyses to cover the whole geographic extent of the northwest Pacific Ocean.

## 2 Data Description

**Fig. 1:**
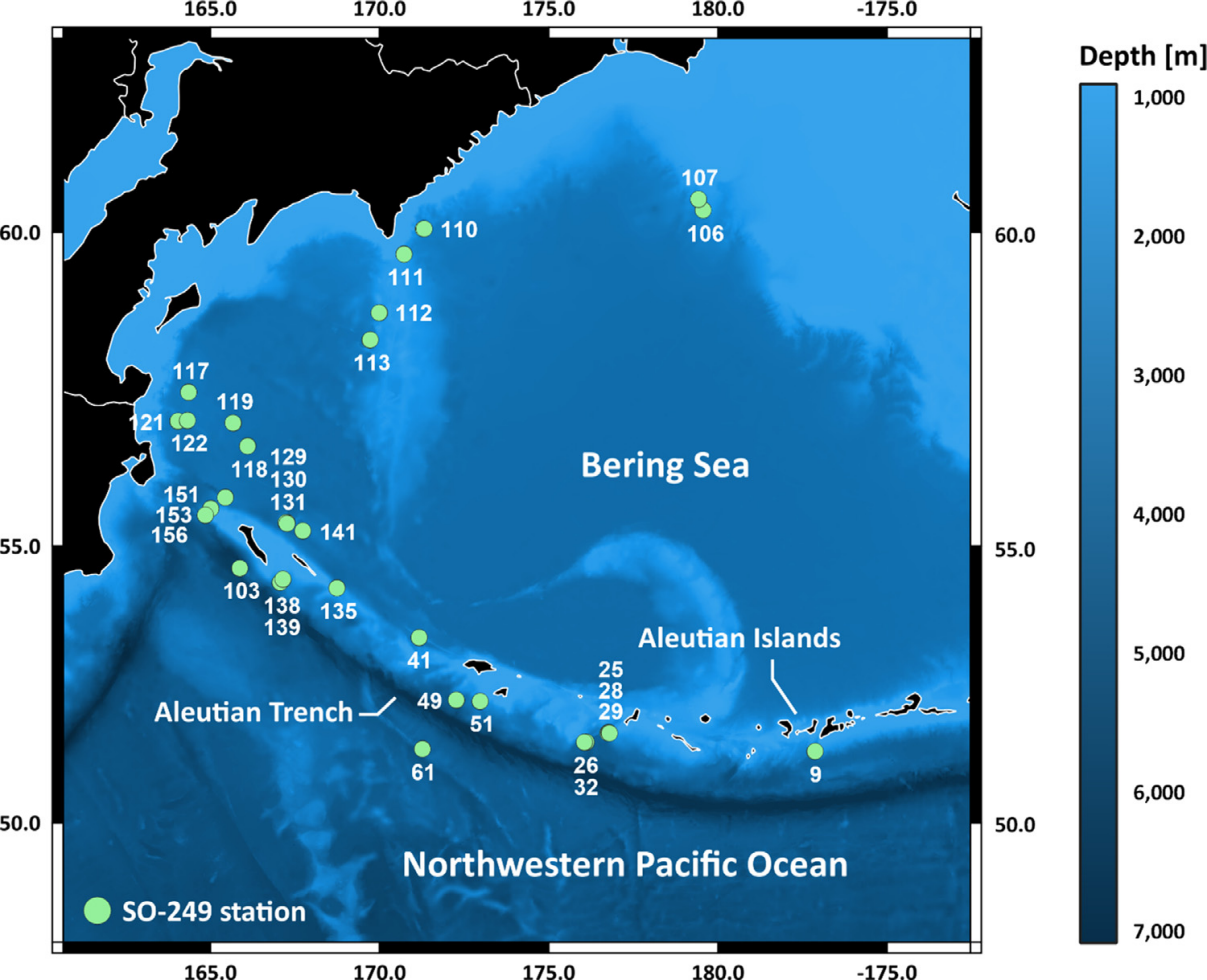
Study area of scientific cruise SO-249 BERING displaying all sampling stations (green circles and numbers) that contained macrofaunal organisms (Crustacea, Polychaeta, Sipuncula, Hirudinea) included in the present dataset.

The herein presented dataset comprises new, unpublished specimen records of Crustacea, Polychaeta, Sipuncula, and Hirudinea from the Bering Sea and the northwestern Pacific Ocean (Fig. 1), collected during scientific cruise SO-249 BERING. All data were formatted based on the Darwin Core standard [1], quality control of the generated data was performed to allow for FAIR data sharing. In total, the dataset comprises 78 unique occurrence records, of which 26 belong to Crustacea, one belongs to Hirudinea, 47 belong to Polychaeta and four belong to Sipuncula. All polychaete records were identified to family level, while only 46.2 % of the crustacean records could be identified to family level or lower ranks (genus, species). The complete occurrence record dataset is available in OBIS [8] and GBIF [9]. These macrobenthic species records are complemented by bathymetric and biogeographic metadata as extracted from their corresponding station data (Table 1). In OBIS the stored data is available in two separate CSV-files, one containing the occurrence data, the other one containing sampling measurement information. The GBIF database offers three different data download CSV-formats: (1) Simple comprises the interpreted data and coordinates; (2) Darwin Core Archive contains raw, interpreted, multimedia and coordinate data; and (3) Species comprises the interpreted data.

**Table 1:** List of stations from scientific cruise SO-249 BERING that contained macrofaunal organisms (Crustacea, Polychaeta, Sipuncula, Hirudinea only). All stations were sampled using a chain bag dredge. F.Z. = Fracture Zone.

Station	Date	Area	Start Coordinates	End Coordinates	Depth [m]	Footprint [WKT]
SO249-009	10.06.2016	Adak Canyon	51° 20.391′ N 177° 08.025′ W	51° 20.735′ N 177° 07.318′ W	2751.5-3322.5	MULTIPOINT (-177.13400 51.34000, -177.12200 51.34600)
SO249-025	16.06.2016	Murray Canyon	51° 41.511′ N 176° 45.374′ E	51° 41.058′ N 176° 45.161′ E	3197.8-3627.5	MULTIPOINT (176.75600 51.69200, 176.75300 51.68400)
SO249-026	16.06.2016	Murray Canyon	51° 30.644′ N 176° 06.580′ E	51° 31.101′ N 176° 06.505′ E	4067.1-4466.6	MULTIPOINT (176.11000 51.51100, 176.10800 51.51800)
SO249-028	17.06.2016	Murray Canyon	51° 41.627′ N 176° 46.882′ E	51° 41.159′ N 176° 46.943′ E	3013.8-3591.0	MULTIPOINT (176.78100 51.69400, 176.78200 51.68600)
SO249-029	17.06.2016	Murray Canyon	51° 41.282′ N 176° 47.343′ E	51° 40.768′ N 176° 47.923′ E	2203.3-2925.0	MULTIPOINT (176.78900 51.68800, 176.79900 51.67900)
SO249-032	18.06.2016	Murray Canyon	51° 30.479′ N 176° 03.454′ E	51° 30.872′ N 176° 03.387′ E	3823.4-4865.3	MULTIPOINT (176.05800 51.50800, 176.05600 51.51500)
SO249-041	21.06.2016	Kresta Ridge	53° 24.325′ N 171° 10.340′ E	53° 24.680′ N 171° 10.363′ E	2818.9-3320.3	MULTIPOINT (171.17200 53.40500, 171.17300 53.41100)
SO249-049	24.06.2016	Attu Canyons	52° 16.917′ N 172° 16.626′ E	52° 17.376′ N 172° 16.215′ E	3306.3-3740.2	MULTIPOINT (172.27700 52.28200, 172.27000 52.29000)
SO249-051	25.06.2016	Aleutian slope SW of Attu	52° 15.599′ N 172° 58.251′ E	52° 15.932′ N 172° 58.612′ E	1112.9-1491.5	MULTIPOINT (172.97100 52.26000, 172.97700 52.26600)
SO249-061	28.06.2016	Stalemate F.Z.	51° 23.629′ N 171° 16.397′ E	51° 23.175′ N 171° 16.113′ E	2518.4-3131.7	MULTIPOINT (171.27300 51.39400, 171.26900 51.38600)
SO249-103	18.07.2016	Komandorsky Block	54° 36.912′ N 165° 52.248′ E	54° 37.304′ N 165° 52.295′ E	4749.8-5070.5	MULTIPOINT (165.87100 54.61500, 165.87200 54.62200)
SO249-106	21.07.2016	Beringia Margin	60° 20.013′ N 179° 33.920′ E	60° 19.685′ N 179° 34.100′ E	2110.9-2511.8	MULTIPOINT (179.56500 60.33400, 179.56800 60.32800)
SO249-107	21.07.2016	Beringia Margin	60° 29.006′ N 179° 25.625′ E	60° 29.340′ N 179° 25.729′ E	1965.1-2309.7	MULTIPOINT (179.42700 60.48300, 179.42900 60.48900)
SO249-110	24.07.2016	Chukotka Margin	60° 03.132′ N 171° 17.789′ E	60° 03.183′ N 171° 19.464′ E	1906.0-2425.3	MULTIPOINT (171.29600 60.05200, 171.32400 60.05300)
SO249-111	24.07.2016	Chukotka Margin	59° 40.733′ N 170° 43.552′ E	59° 40.247′ N 170° 43.640′ E	1204.7-1735.6	MULTIPOINT (170.72600 59.67900, 170.72700 59.67100)
SO249-112	24.07.2016	Shirshov Ridge	58° 47.112′ N 170° 00.254′ E	58° 47.074′ N 169° 59.361′ E	1443.4-1907.8	MULTIPOINT (170.00400 58.78500, 169.98900 58.78500)
SO249-113	25.07.2016	Shirshov Ridge	58° 21.666′ N 169° 43.046′ E	58° 22.040′ N 169° 43.764′ E	2283.1-2741.8	MULTIPOINT (169.71700 58.36100, 169.72900 58.36700)
SO249-117	26.07.2016	Beta F.Z.	57° 32.585′ N 164° 21.446′ E	57° 32.228′ N 164° 21.562′ E	2768.3-2907.8	MULTIPOINT (164.35700 57.54300, 164.35900 57.53700)
SO249-118	26.07.2016	Beta Rise, Beta F.Z.	56° 40.456′ N 166° 06.409′ E	56° 39.990′ N 166° 06.058′ E	3270.8-3519.6	MULTIPOINT (166.10700 56.67400, 166.10100 56.66700)
SO249-119	27.07.2016	Beta Rise, Beta F.Z.	57° 02.478′ N 165° 40.930′ E	57° 02.749′ N 165° 40.217′ E	3190.8-3456.5	MULTIPOINT (165.68200 57.04100, 165.67000 57.04600)
SO249-121	27.07.2016	Alpha F.Z.	57° 04.196′ N 164° 02.374′ E	57° 04.660′ N 164° 02.829′ E	2954.1-3261.8	MULTIPOINT (164.04000 57.07000, 164.04700 57.07800)
SO249-122	28.07.2016	Beta Rise, Alpha F.Z.	57° 04.798′ N 164° 19.204′ E	57° 05.245′ N 164° 19.591′ E	2430.6-2757.4	MULTIPOINT (164.32000 57.08000, 164.32700 57.08700)
SO249-129	30.07.2016	Piip volcano	55° 23.719′ N 167° 16.286′ E	55° 23.983′ N 167° 16.136′ E	644.2-894.8	MULTIPOINT (167.27100 55.39500, 167.26900 55.40000)
SO249-130	30.07.2016	Piip volcano	55° 23.736′ N 167° 14.170′ E	55° 23.947′ N 167° 14.312′ E	906.8-1147.7	MULTIPOINT (167.23600 55.39600, 167.23900 55.39900)
SO249-131	30.07.2016	Piip volcano	55° 23.072′ N 167° 16.242′ E	55° 22.900′ N 167° 15.944′ E	539.1-707.7	MULTIPOINT (167.27100 55.38500, 167.26600 55.38200)
SO249-135	31.07.2016	Guyot SE of Medny Island	54° 17.007′ N 168° 44.712′ E	54° 16.542′ N 168° 44.567′ E	330.7-896.4	MULTIPOINT (168.74500 54.28300, 168.74300 54.27600)
SO249-138	01.08.2016	Komandorsky Block	54° 22.475′ N 167° 03.577′ E	54° 22.699′ N 167° 04.127′ E	1049.9-1419.3	MULTIPOINT (167.06000 54.37500, 167.06900 54.37800)
SO249-139	01.08.2016	Komandorsky Block	54° 25.640′ N 167° 08.953′ E	54° 26.065′ N 167° 08.742′ E	432.2-901.5	MULTIPOINT (167.14900 54.42700, 167.14600 54.43400)
SO249-141	02.08.2016	Bathymetric height SE of Piip Volcano	55° 15.260′ N 167° 43.923′ E	55° 15.632′ N 167° 44.018′ E	3608.0-3823.2	MULTIPOINT (167.73200 55.25400, 167.73400 55.26100)
SO249-151	05.08.2016	Komandorsky Block	55° 49.600′ N 165° 26.784′ E	55° 49.125′ N 165° 26.662′ E	3178.6-3505.1	MULTIPOINT (165.44600 55.82700, 165.44400 55.81900)
SO249-153	05.08.2016	Komandorsky Block	55° 38.307′ N 165° 00.704′ E	55° 37.987′ N 165° 00.766′ E	1904.2-2163.9	MULTIPOINT (165.01200 55.63800, 165.01300 55.63300)
SO249-156	06.08.2016	Komandorsky Block	55° 31.146′ N 164° 51.149′ E	55° 31.553′ N 164° 51.329′ E	2419.3-2928.2	MULTIPOINT (164.85200 55.51900, 164.85500 55.52600)

## 3 Experimental Design, Materials and Methods

In 2016, the scientific cruise SO-249 BERING explored the deep-sea of the western Bering Sea, the northwestern Pacific Ocean, as well as the area surrounding the Aleutian Islands and Aleutian Trench using deep-sea RV *Sonne* [2].

Rectangular chain bag dredges were used to conduct geological and biological sampling. A total of 150 dredge hauls, of which 112 contained macrofaunal organisms, yielded about 1,500 macrofaunal specimens that were deposited in the collections of the Museum für Naturkunde in Berlin, Germany [2]. All specimens were preliminarily sorted and selected megafaunal specimens (Cephalopoda: Octopoda) were analysed in-depth [10]. Those samples belonging to either Crustacea, Polychaeta, Sipuncula or Hirudinea were morphologically identified to the lowest taxonomic rank possible based on taxonomic keys and literature [11], [12], [13] using a Leica M60 stereomicroscope. Subsequently, the specimen record dataset was prepared based on the Darwin Core format [1] for submission to the OBIS [8] and GBIF [9] open-access databases, including detailed biogeographic metadata as extracted from the cruise report [2].

## CRediT authorship contribution statement

**Henry Knauber:** Investigation, Data curation, Writing – original draft. **Katharina Kohlenbach:** Investigation, Data curation. **Philipp Böhm:** Investigation. **Carsten Lüter:** Project administration, Resources. **Alexander Ziegler:** Project administration, Resources. **Angelika Brandt:** Conceptualization, Writing – review & editing, Supervision, Project administration, Funding acquisition. **Hanieh Saeedi:** Conceptualization, Data curation, Writing – review & editing, Visualization, Supervision, Project administration, Funding acquisition.

## Declaration of Competing Interest

The authors declare that they have no known competing financial interests or personal relationships that could have appeared to influence the work reported in this paper.

## Data Availability

Deep-sea benthic crustacean, polychaete, and sipunculid data from the Bering Sea (Original data) (GBIF, OBIS).
